# Managing and monitoring a pandemic: showcasing a practical approach for the genomic surveillance of SARS-CoV-2

**DOI:** 10.1093/database/baad071

**Published:** 2023-10-16

**Authors:** Mateusz Jundzill, Riccardo Spott, Mara Lohde, Martin Hölzer, Adrian Viehweger, Christian Brandt

**Affiliations:** Institute for Infectious Diseases and Infection Control, Jena University Hospital, Erlanger Allee 103, Jena 07747, Germany; Leibniz Center for Photonics in Infection Research (LPI), Albert-Einstein-Str. 9, Jena 07747, Germany; Institute for Infectious Diseases and Infection Control, Jena University Hospital, Erlanger Allee 103, Jena 07747, Germany; Institute for Infectious Diseases and Infection Control, Jena University Hospital, Erlanger Allee 103, Jena 07747, Germany; Methodology and Research Infrastructure, Genome Competence Center (MF1), Robert Koch Institute, Seestraße 10, Berlin 13353, Germany; Institute of Medical Microbiology and Virology, University Hospital Leipzig, Johannisallee 30, Leipzig 04103, Germany; Institute for Infectious Diseases and Infection Control, Jena University Hospital, Erlanger Allee 103, Jena 07747, Germany; Leibniz Center for Photonics in Infection Research (LPI), Albert-Einstein-Str. 9, Jena 07747, Germany; InfectoGnostics Research Campus, Philosophenweg 7, Jena 07743, Germany

## Abstract

With the rapidly growing amount of biological data, powerful but also flexible data management and visualization systems are of increasingly crucial importance. The COVID-19 pandemic has more than highlighted this need and the challenges scientists are facing. Here, we provide an example and a step-by-step template for non-IT personnel to easily implement an intuitive, interactive data management solution to manage and visualize the high influx of biological samples and associated metadata in a laboratory setting. Our approach is illustrated with the genomic surveillance for SARS-CoV-2 in Germany, covering over 11 600 internal and 130 000 external samples from multiple datasets. We compare three data management options used in laboratories: (i) simple, yet error-prone and inefficient spreadsheets, (ii) complex and long-to-implement laboratory information management systems and (iii) high-performance database management systems. We highlight the advantages and pitfalls of each option and outline why a document-oriented NoSQL option via MongoDB Atlas can be a suitable solution for many labs. Our example can be treated as a template and easily adapted to allow scientists to focus on their core work and not on complex data administration.

## Introduction

### Managing a high sample influx

The start of the COVID-19 pandemic has created an unprecedented global genomic surveillance effort through whole genome sequencing (WGS) to monitor the emergence and evolution of the causing agent, SARS-CoV-2, on a larger scale. To put this into perspective, the international Global Initiative on Sharing Avian Influenza Data (GISAID; https://www.gisaid.org/) collected over 15.84 million SARS-CoV-2 whole genome sequences (as of August 2023). To better coordinate and support these sequencing efforts, the German government signed the Coronavirus Surveillance Regulation (CorSurV) (1), which is comparable to initiatives from other governments such as COG-UK (United Kingdom) (2) or INSA-COG (India) (INSA-COG; https://www.pib.gov.in/PressReleseDetailm.aspx?PRID=1684782) and compensated laboratories for sequencing SARS-CoV-2-positive samples and submitting the resulting genomes to the Robert Koch Institute (RKI), Germany’s national Public Health institute, for nationwide genomic surveillance. As part of this regulation, the Jena University Hospital sequenced collected samples for one of the states in Germany (Thuringia) and submitted the analyzed genomes to the RKI via the ‘German Electronic Sequence Data Hub’ (GISAID; https://www.rki.de/DE/Content/InfAZ/N/Neuartiges_Coronavirus/DESH/DESH.html). We routinely sequence approximately 200 SARS-CoV-2-positive samples per month, which is a 10-fold increase from the start of the project in early 2020 (MongoDB Charts dashboard; https://bit.ly/3MtK2v2). We managed (as of January 2023) over 140 000 samples, including 11 600 sequenced in-house, gathered from cooperation partners and GISAID samples from Germany collected for research purposes. The sheer number of samples makes it increasingly difficult to access the data by normal means or to verify the overall sequencing progress per sample, the integrity of the data, its origin and storage location in the laboratory, while enforcing a uniform scheme. To improve our data management strategies, we were looking for a solution that is simple to learn and flexible, capable of storing multiple datasets and metadata. It should also be data source agnostic, allowing for data import from multiple sources, and accepting universal data formats. At the same time, it was essential to maintain ease of use for technical laboratory assistants, researchers, external partners and clinicians while quickly identifying and preventing data errors or mistakes.

Similar needs and difficulties were observed in managing data in the mids of the Ebola epidemic in Sierra Leone (2014/2015). These provided valuable parallels to the observations and challenges in managing epidemic data during the SARS-CoV-2 pandemic. During the early stages of both outbreaks, data loss was a significant problem due to inexperienced personnel, the absence of a centralized database and uniform input files leading to oftentimes unnoticed mistakes that contributed to significant data loss (3). Likewise, during the SARS-Cov-2 pandemic in Ghana, the personnel was overwhelmed by relying on manual systems, primarily based on spreadsheets that forced them to transition toward more advanced and automated database systems to streamline data management processes.

The authors also emphasize the importance of integrating data from various devices, such as polymerase chain reaction (PCR) machines, showing the usefulness of using a standard, non-propertiary data format. In their example, the PCR machine output was exported to a spreadsheet and integrated by the integration team with the metadata for swift data merging. Another challenge highlighted in the study relates to separating data for the various stakeholders from the original dataset, which was also solved by a proper database system. Furthermore, the study underscores the significance of visualizing geographical coordinates to identify potential epidemiological hotspots and to have a visual overview of all the data. Geographical visualization tools are essential to improve the detection and response to disease outbreaks by pinpointing areas with increased transmission risks (4). After considering various data management systems, previous experiences and our needs, we decided on a document-oriented NoSQL database management system to manage biological samples and continuously provide our research institute and external partners with aggregated data and results.

### Choosing a suitable data management system

We considered three suitable and popular options to manage biological samples: spreadsheets, laboratory information management systems (LIMS) and database management systems (DBMS). Based on our previous experience, we considered the following criteria to be highly important:

- Difficulty: it should be easy to set up and maintain
- Performance: the ability to handle thousands of samples and data requests
- Accessibility: should be able to be operated with various computer skills and provide role management to separate read, write and administrative rights
- Error management: robust and easy system to spot spelling mistakes or incorrect data with data backup options and corruption protection
- Scalability/Flexibility: the possibility for vertical and horizontal scale and the capability of accepting a new type of data after the initial set up
- Security: the system should be able to handle sensitive and confidential data and protect from unauthorized access
- Maintenance cost: low time and financial cost of the system maintenance

### Spreadsheets

The widely used and simplest approach for handling data in a laboratory is the spreadsheet component of an office productivity suite (e.g. Microsoft Excel, Google Sheets, LibreOffice Calc). Spreadsheets are commonly used in research to store, organize, analyze and plot data quickly. Data in a spreadsheet are stored in a tabular format, ideally with each row containing a single record and each column an attribute describing the record (long spreadsheet format). The unexpected downside of its simplicity is that people who are not experienced with data storage good practices may prioritize the appearance of the data over data accessibility and transferability, e.g. color coding or storing multiple datasets in one spreadsheet. Moreover, spreadsheets are unsuitable for large datasets due to bad performance and navigation problems. The absence of an indexed, unique ID system leads to data duplication, and the lack of advanced data validation makes it hard to detect typographical and formatting errors. Another downside is how easily data records can be overwritten, exchanged or misaligned. These problems can arise even without user interference due to the implemented autocorrection of office suites, like removing leading zeros or auto-converting data to a calendar or time format. Data corruption is so problematic that Ziemmann *et al.* found gene name formatting errors in 704 of 3597 screened supplementary data sets from published articles (5). Furthermore, a proprietary compressed format (.xls, .xlsx, .ods) may be inconsistent between different software versions.

Despite all these disadvantages, Public Health England used Microsoft Excel to store their SARS-CoV-2 data and lost 16 000 entries in 2020 due to the maximum row limit in Microsoft Excel (6).

Collaborative work on spreadsheets is problematic as the entire file must be shared, which usually results in multiple file versions being in circulation and requiring laborious and error-prone merging. While cloud solutions like Google Calc address this problem, they might be ill-suited for sensitive information (7). While spreadsheets are a useful, simplistic tool to support data administration, they are the least favorable solution for continuous data storage.

### Laboratory information management systems

An alternative solution is LIMS. LIMS are software applications designed to centralize and assist in managing laboratory data, combining all laboratory devices and processes in one integrated large system (8). As an analogy, LIMS performs a similar function to business management software like Enterprise Resource Planning (e.g. SAP) that is used to manage company operation and integrate company departments. Generally, the software allows users to register data and laboratory equipment, manage orders and provide the functionality of an electronic lab book. LIMS vary from one another and can be tailor-made or adjusted to suit the needs of a company or institution. The scalability and performance of LIMS depend on the chosen solution. The main advantage of LIMS is the automation of routine tasks (e.g. results archiving, data sharing or stock monitoring) that it provides. LIMS typically feature a web-based interface that allows users to easily access the data and instruments. The ID tracking system, often combined with a barcode reader, removes data duplication problems. Moreover, automatic record update through connected laboratory instruments reduces the time and the risk of erroneous data input by personnel. Personal logins, passwords and data encryption enforce data security, role protection and secure access at every access point in the network (9, 10). Depending on the chosen system or service, data backup could be handled on an external or local server by a software provider. LIMS can be complex and costly to implement and the deep integration of LIMS into the laboratory network causes slow implementation, requires specialized IT personnel to maintain the system and training for the everyday user. Due to the aforementioned time consumption and cost, LIMS might only be suited for some, mostly large institutions.

### Database management systems

The middle ground between the simplicity of a spreadsheet and the complexity of a LIMS is database management system. A database management system (DBMS) is a software for creating and managing databases. With such a program, users can enter data into a database to collect, read, update and delete large amounts of data. These systems can be adapted to any kind of data, like spreadsheets, but are optimized for storing and retrieving large datasets while providing access control mechanisms to ensure data security and integrity, redundancy control (ID assignment), data auditing and optimization for collaborative work. Additional benefits include crash protection (database transaction system based on commits), in-depth user-defined security options (different security protocols, data encryption, registering user actions in a separate file, real-time usage monitoring) and data backup. In this article, we considered two of the most commonly used groups of database management systems: Relational Databases (RDBMS) and Non-Relational NoSQL stores/DBMS.

Relational databases are the most common type of DBMS and are well suited for storing structured data. Modern RDBMS use the Structured Query Language (SQL) to access, query and modify data that are stored in a relational table format. RDBMS enforces a predefined schema that needs to be assumed before the database creation, thus providing limited support for unstructured data compared to the latter group. This often results in empty or underutilized fields (11). The rigid data structure can make it complicated to accommodate changing data needs or create a less clear data structure (12).

The other group, NoSQL DBMS, is a non-uniform collection of database management systems/stores that are not based on the traditional RDBMS model. Unlike the former group of DBMS, each NoSQL store has distinct characteristics and functionalities, making them unique systems with their own data models. As a result, there is no uniform data model across all NoSQL stores, and they differ significantly from one another. Yet, most NoSQL databases are valued for their flexibility and scalability (13). Unlike RDBMS, NoSQL stores are usually schemaless, meaning they do not require predefined and non-changing data structures at the cost of performance. The data can be stored in various structures like a collection of documents (MongoDB), graphs (Neo4J), key-values (Redis) or wide columns (Apache Cassandra) (14). In recent years, MongoDB emerged as one of the most popular and widely used NoSQL stores. Thus, various online guides, the integration of multiple tools and a supportive community are available. Moreover, an extensive ecosystem is provided by MongoDB, especially for their cloud-based solution. It offers a user-friendly web browser interface, multiple server locations to comply with local data protection regulations. Graphical user interface (GUI) tool—MongoDB Compass— allows interaction with the database in a user-friendly way. Furthermore, the most crucial point is the availability of an online visualization tool—MongoDB Charts—which provides visual real-time data surveillance. This customizable data surveillance reduces data administration complexity, offers external stakeholders faster and aggregated data access and removes the need to transfer data to other external visualization tools.

## MongoDB

### Description and structure of MongoDB

**Figure 1.**
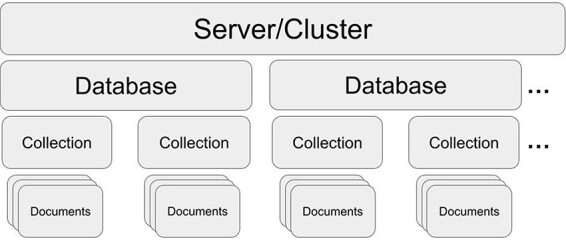
MongoDB structure. One server/cluster can store several databases. Each database can contain collections that store multiple and separate records as documents (in JSON style).

MongoDB is an open-source, document-oriented, NoSQL database management system that has been actively developed since 2009 (MongoDB; https://www.mongodb.com). MongoDB stores data in collections of JSON-like documents (see section ‘MongoDB document’ and Figure 1). It is available as an open source, community edition, and as a commercial distribution that includes advanced security and administration methods. It is also offered in a standalone, self-administered, deployed local DBMS and a cloud-hosted database-as-a-service variant (MongoDB Atlas). MongoDB Atlas simplifies the complexities of deploying, managing and scaling MongoDB DBMS without the need to buy additional hardware. Moreover, the online version by default is replicated on three servers (shards), forming a cluster and automatic failover, providing an additional layer of redundancy. Despite the fundamental difference between storing data in tabular and document format, it is possible to find structural analogs to a typical spreadsheet format (see section ‘MongoDB document’). A document is self-describing and schemaless, providing complete flexibility. Documents contain information in a ‘key: value’ format for each document row. The ‘key’ serves as a unique identifier like a category that is used to aggregate and retrieve data that is stored as a ‘value’ (e.g. Species: ‘Staphylococcus aureus’). This enables robust and readable data entry. Keys do not have to be present in all documents, in contrast to RBMS-based solutions, where keys are predefined across all samples.

Setting up an online instance of Mongo does not require extensive IT knowledge and can be done exclusively via a GUI. The initial build, creating the first user, and populating the database with prepared data in a CSV (comma-separated values) spreadsheet format takes approximately 30 minutes using the web browser interface of the Mongo Atlas website. This approach is hardware agnostic and relies solely on the internet connection. The data input can be done from a JSON file (structured text format), a CSV spreadsheet import or directly via the GUI. The import of data from spreadsheets is especially useful for personnel who do not have advanced programming expertise. Moreover, when regulations prohibit data access from outside of the institution, the database can be deployed in an internal network or on a selected local machine. However, it needs to be stressed that a local deployment requires more IT expertise and time, as all the security and data integrity steps are not automated and depend on the data administrator. Therefore, it is not recommended for beginners. In cases where only a portion of data should be accessed from external networks, Mongo Sync allows the synchronization of local and online databases.

Further database customization options are also available, providing administrators with user-written scripts. These scripts allow for more advanced automatic data manipulation or backups (e.g. auto-correction of common mistakes, enforcing a uniform data structure and creating additional variables based on previously entered data).

### MongoDB document

**Figure 2.**
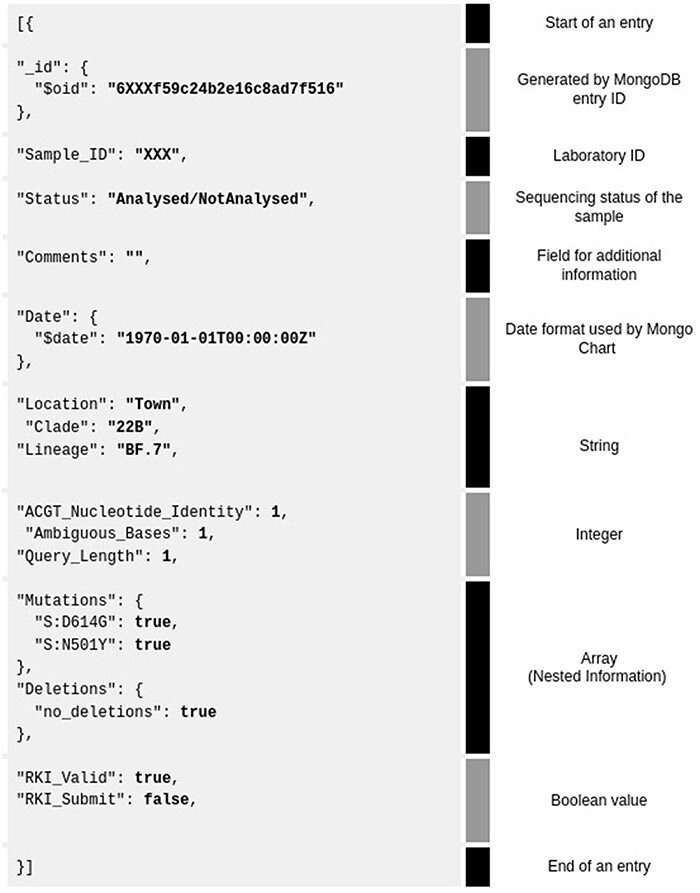
Example of a MongoDB JSON database entry for one sample (grey background). Shown are all the 3–4 data types and their corresponding explanation on the right. The database entry is separated by category (text in black, called ‘key’) and value of data (bold—‘value’) for visualization purposes.

MongoDB documents have a structure of JSON-like files (Figure 2). A MongoDB JSON data point has a structure of ‘key’: ‘value’, separated from each other by a comma. The database can be populated directly from a JSON file or a CSV spreadsheet. However, CSV import does not allow array (nested) data entry or distinguishing between data types.

Numerical values are written without quotes. The ‘_id’ field is recognized by the MongoDB document identification field that allows future updating of a document. An example of an array that contains multiple key:value records under one general key are ‘Mutations’ and ‘Deletions’ keys (Figure 2).

### Visualization of data and results

Data visualization and sharing for biological samples can be accomplished through standalone tools such as Nextstrain (https://www.nextstrain.org) and Microreact (https://www.microreact.org). These platforms allow uploading and visualizing genomic datasets through a web-based application and sharing them with URLs. However, these tools require tool-specific dataset and adjusted metadata formats for the visualization. Additionally, they can only update the data with new records, by an entire dataset resubmit, limiting efficient real-time data monitoring. Moreover, the separate platform adds further complexity and additional data administration to the preexisting data solution.

**Figure 3.**
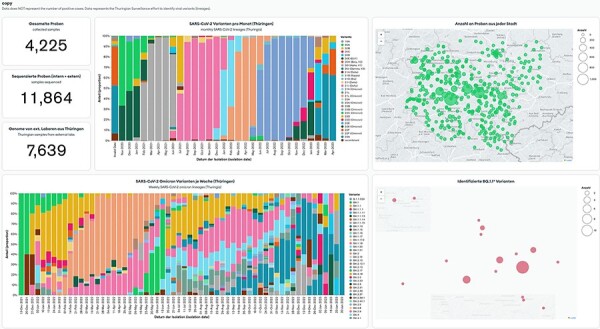
MongoDB Charts dashboard example based on the Thuringian Surveillance Effort for SARS-CoV-2 genomic sequencing. MongoDB Charts are accessible at https://www.bit.ly/3MtK2v2.

To address these challenges, we propose an alternative solution that uses an integrated MongoDB Atlas tool—MongoDB Charts. MongoDB Charts is a self-updating data visualization tool to present records from the MongoDB Atlas database. MongoDB Charts works in a drag-and-drop fashion by selecting desirable graphs and fields from any collection or database stored on a cluster. The provided data points can be filtered and modified via the aggregation feature without changing the data. These allow conditionally selecting a part of the data, e.g. for a particular period or below a cutoff value, doing mathematical manipulations to change units, or combining data from multiple fields. MongoDB Charts allows users to generate standard charts like bar-, line- and pie charts, numeric figures, tables and map charts (Figure 3). The latter, in particular, allows the real-time display of results stored in the database on one or more maps for surveillance purposes. Online dashboards store the created plots; the user can arrange multiple charts together and share them online on a user access principle or publicly. Multiple dashboards can be created to present data for different audiences (e.g. external partners, laboratory personnel, medicine practitioners). Furthermore, the MongoDB Charts visualization method is vital for data exploration, pattern recognition and spotting potential errors quickly.

## Implementation of MongoDB for biological samples

### Setup

In the following steps, we present a simplified description of how to set up MongoDB for a laboratory environment using the web interface of Mongo Atlas and how to manage the database with a locally installed Mongo Compass (MongoDB Compass; https://www.mongodb.com/try/download/compass). To make this process as straightforward as possible, we additionally provide a step-by-step protocol (15) and GitHub repository (Routine Seq SARS-CoV-2 MongoDB Database; https://github.com/AggresiveHayBale/Routine-Seq-SARS-CoV-2-MongoDB-Database) with useful commands and curation scripts that could be readapted for other purposes. The online protocol and the repository will be updated in case of future changes in the process. Firstly, we create the cluster (see Figure 1 for an overview of the MongoDB structure) directly via the MongoDB Atlas website (MongoDB Atlas; https://www.mongodb.com/atlas/database), after creating a user account and a project (e.g. one project per institute or working group). We suggest selecting a free shared cluster tier that is optimal for a small-scale deployment in a research institution. The server provider and location could be selected based on the preferred data protection policies of the user’s country of origin or the location of an institution.

Secondly, create a database and a collection on the server from Mongo Compass. The database and collection name should not use any special characters (like ‘$,(,§,%’ or non-standard English characters) and be as concise as possible. The server/cluster can be accessed with Mongo Compass by using the provided connection string, which is given during cluster creation (‘mongodb+srv://<username>:<password>@<hostname>.net/’).

This completes the initial setup, and the database can now be filled with data, e.g. by importing a CSV file.

At the beginning of implementing MongoDB in an institution, we suggest keeping the already established method of data archiving (e.g. spreadsheets) still operational to reduce potential damage done to the data by inexperienced users. Additional advanced functions can be added after getting more accustomed to the basic functionality. The administrator should verify default settings in the security tab to limit network access and create user profiles with adequate roles. There are three predefined major roles: ‘Read’, which can only view the database but cannot modify documents, ‘Read/Write’, which can view and modify entries and ‘Admin’, which have read/write permissions but can also modify the database settings. To avoid potential security infringements and improve data integrity, the lowest possible role should be given to the user (e.g. not giving a read/write role to personnel that does not update the database) and avoid using the admin role for routine work.

It is important to add a database backup for data security or recovery purposes. The quick exporting of a full database in Mongo Compass to a JSON/CSV file would not fully keep the integrity of the data and is not a sustainable, long-term solution. The Mongo Dump command available from Mongo Tools toolkit provides a better approach that allows restoration or replication of the database without such problems. Alternatively, higher cluster tiers provide automatic backup plans administered by the MongoDB company.

Data auditing can be performed from Mongo Compass’ Schema tab, which gives insight into a data structure and allows to catch common mistakes like wrongly entered numerical data, alternative spelling or text formatting (e.g. München, Munchen, munchen, Munich). In the most naive approach, the data correction can be done by manual value change in the database, updating entries with new values from JSON/CSV files.

**Figure 4.**
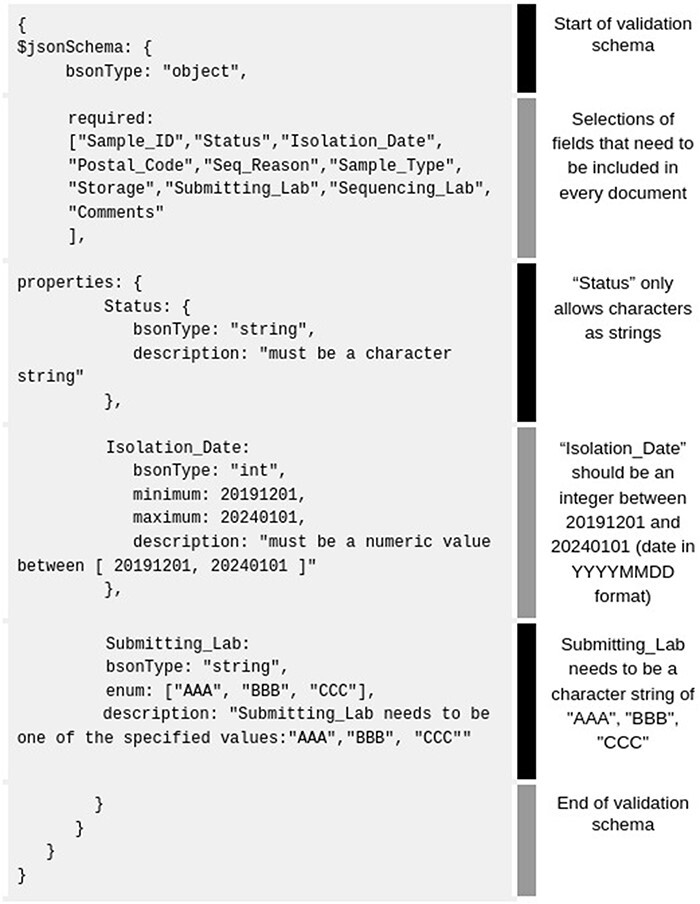
Example of basic MongoDB schema rules (gray background) accepted by the MongoDB Compass validation tab. For better clarity, we added our own fields like ‘Status’, ‘Isolation _ Date’, ‘Submitting_ Lab’, etc.

Non-manual data curation can be performed either by using custom-made scripts that will correct common mistakes or by enforcing strict data integrity checks via predefined schema. Schema rules can be directly set from the MongoDB Atlas website or through MongoDB Compass (validation rules, see Figure 4).

### From sample to database entries (example: SARS-CoV-2 genomic surveillance)

In the following part, we present a shortened example of a DBMS-driven data management as a standard operating procedure (SOP) for SARS-CoV-2 genomic surveillance that can be adjusted to any biological sample or lab procedure. Additionally, we provide a more extensive version as a version-controlled online supplement (16). Our methodology uses a CSV document for data import, allowing the protocol to remain independent of any specific method. This flexibility enables the application of our approach to update data from diverse sources, including machine-generated reports from e.g. PCR machines—the current dominant form of SARS-CoV-2 surveillance. Many of the steps can be changed but we went with a CSV export/import procedure to allow easy data manipulation for technical lab assistants without any database knowledge. Importing the initial CSV can be further simplified by using an online Google Forms survey (Google Forms; https://forms.google.com/) that is connected to an online Google Calc spreadsheet. This approach can work as an additional level of data curation, as an option to centralize data import to the database or if additional software like MongoDB Compass cannot be installed.

1. Sample(s) arrives for DNA sequencing from an internal or external partner
2. Based on the information provided by the partner, a basic sample entry is created from a template spreadsheet CSV. The template contains fields like Sample_ID, Status, Isolation_Date, Postal_Code, Seq_Reason, Sample_Type, Storage, Submitting_Lab, Sequencing_Lab and Comments in the header of the CSV
3. The CSV is filled out and imported to the database using MongoDB Compass application or via the command line tool (Mongoimport)
4. The prepared input is verified and adjusted to a standardized schema
5. The successful upload is confirmed. If entered data offend the previously established scheme, the upload would fail (e.g. postal code containing letters or status containing a different value than predefined)
6. Data input is verified using MongoDB Compass or MongoDB Charts dashboard plots (e.g. erroneous geographical points on the map, unusual data values). Optionally custom-made scripts can be executed to detect these inconsistencies or add additional information based on the other fields
7. Laboratory personnel checks and selects samples in MongoDB Compass for sequencing by querying the database with e.g.: {Status: ‘not sequenced’}
8. The Status field can then be changed to e.g. the name of the person or ‘being processed’ to mark the sample as being processed for others
9. A CSV can then be exported to modify later and printed for the lab to work with. A CSV can be exported by using the {Status:{‘being processed’}} in the search bar and then exported as CSV
10. Samples that are sequenced and or analyzed can be updated in the exported CSV and then import the new information to the DB

## Conclusion

The increasing amount of data and metadata collected by medical and research facilities require more comprehensive and efficient solutions for data storage. Traditional solutions like spreadsheet files are too cumbersome and do not work after exceeding a certain amount of data. At the same time, other solutions like laboratory management software and relational databases are too complex and time-consuming to implement and maintain for most people. MongoDB is an efficient and flexible tool for managing biological samples and data. It offers a range of features that make it well suited for managing large data collections. It includes a user-friendly graphical user interface, the ability to easily store and retrieve data, scalability and security. One of the most crucial elements of surveillance, the process of delivering and communicating data, is streamlined by integrating an automatically updated visualization tool, thus removing the need for additional software maintenance and providing direct, hassle-free access to the results. We provide an easy-to-read, use and adapt template for implementing MongoDB into routine laboratory data management that allows scientists to focus on their core work and not on complex data administration. The example uses genomic sequencing data to demonstrate the protocols in a more challenging complex dataset. Our approach also applies to other types of biological data (e.g. PCR, antibiotic susceptibility testing) with less intricate internal structures.
